# Ιnterleukin-17A-Enriched Neutrophil Extracellular Traps Promote Immunofibrotic Aspects of Childhood Asthma Exacerbation

**DOI:** 10.3390/biomedicines11082104

**Published:** 2023-07-26

**Authors:** Maria Ntinopoulou, Dimitrios Cassimos, Eugenia Roupakia, Evangelos Kolettas, Maria Panopoulou, Elpis Mantadakis, Theocharis Konstantinidis, Akrivi Chrysanthopoulou

**Affiliations:** 1 Laboratory of Molecular Immunology, Department of Biological Applications and Technology, School of Health Sciences, University of Ioannina, 45110 Ioannina, Greece; marntinop@yahoo.gr (M.N.); theoxari_ko@yahoo.gr (T.K.); 2 Department of Pediatrics, University General Hospital of Alexandroupolis, Democritus University of Thrace Medical School, 68100 Thrace, Greece; dkasimos@med.duth.gr (D.C.); emantada@med.duth.gr (E.M.); 3 Laboratory of Biology, School of Medicine, Faculty of Health Sciences, University of Ioannina, 45110 Ioannina, Greece; ev.roupakia@uoi.gr (E.R.); ekoletas@uoi.gr (E.K.); 4 Biomedical Research Institute, Foundation for Research and Technology-Hellas, 45110 Ioannina, Greece; 5 Department of Microbiology, University General Hospital of Alexandroupolis, Democritus University of Thrace Medical School, 68100 Thrace, Greece; mpanopou@med.duth.gr

**Keywords:** asthma, children, neutrophils, neutrophil extracellular traps (NETs), interleukin (IL)-17A, inflammation, fibroblasts, tissue fibrosis, asthma exacerbation

## Abstract

Childhood asthma is a chronic inflammatory airway disorder that can drive tissue remodeling. Neutrophils are amongst the most prominent inflammatory cells contributing to disease manifestations and may exert a potent role in the progression of inflammation to fibrosis. However, their role in asthma exacerbation is still understudied. Here, we investigate the association between neutrophil extracellular traps (NETs) and lung fibroblasts in childhood asthma pathophysiology using serum samples from pediatric patients during asthma exacerbation. Cell-based assays and NETs/human fetal lung fibroblast co-cultures were deployed. Increased levels of NETs and interleukin (IL)-17A were detected in the sera of children during asthma exacerbation. The in vitro stimulation of control neutrophils using the sera from pediatric patients during asthma exacerbation resulted in IL-17A-enriched NET formation. The subsequent co-incubation of lung fibroblasts with in vitro-generated IL-17A-enriched NETs led fibroblasts to acquire a pre-fibrotic phenotype, as assessed via enhanced CCN2 expression, migratory/healing capacity, and collagen release. These data uncover the important pathogenic role of the NET/IL-17A axis in asthma exacerbation, linking lung inflammation to fibroblast dysfunction and fibrosis.

## 1. Introduction

Pediatric asthma is one of the most common childhood chronic inflammatory disorders and is a serious public health problem. Even though its global prevalence, morbidity and mortality have increased over the last 40 years [1], the disorder can still be considered underdiagnosed and undertreated.

Asthma is characterized by exacerbation episodes with wheezing, coughing, chest tightness and shortness of breath. The disease pathophysiology includes mucus hypersecretion, inflammation and edema of the airway mucosa and, subsequently, bronchospasm [2]. The current well-documented view is that asthma is a heterogenous disease, and hence, it can be considered an “umbrella” term for distinct clinical phenotypes. In other words, a comprehensive understanding of the phenotypic variations of airway-inflammation-dependent asthma (e.g., neutrophilic, eosinophilic) may give rise to more personalized therapeutic approaches for severe asthmatics [3]. Additionally, the data indicate that the disease severity is associated with the sputum neutrophil counts in both adults and children [4,5]. In this context, studies involving asthmatic children have highlighted the key role of neutrophils and neutrophil cytokines in airway inflammation during asthma exacerbation [6].

Furthermore, circulating cells (e.g., eosinophils, macrophages, neutrophils) as well as structural and functional lung cells (e.g., epithelial cells, fibroblasts) can secrete numerous inflammatory mediators contributing, either directly or indirectly, to airway remodeling in asthma [7]. The data have indicated that transforming growth factor beta (TGF-b), which is mainly secreted by eosinophils, influences the activation profile of fibroblasts and the subsequent production of collagen [8,9]. Moreover, it was suggested that interleukin (IL)-17A is an important contributor to the pathogenesis of airway fibrosis. Elevated levels of IL-17A have been detected in bronchoalveolar lavage (BAL), blood and sputum, both in adults and children, and may be positively correlated with the disease severity [10,11,12,13].

Neutrophils represent the most abundant innate immune cell population and are the first to migrate to sites of tissue inflammation [14,15]. Recent data have emphasized that neutrophils are characterized by significant functional and phenotypic plasticity [16], since they are able to rapidly phenotypically and functionally adapt in response to a plethora of disease and environmental cues [17,18]. Neutrophil plasticity is mainly reflected in the most recently described neutrophilic mechanism, known as neutrophil extracellular traps (NETs). During NETs, neutrophils unfold their DNA into the extracellular space, in the form of a mesh of extruded chromatin fibers lined with various highly bioactive neutrophil-derived granular and cytosolic proteins. Although NETs were initially described as anti-microbial defense mechanisms, numerous publications have emphasized their fundamental role in the pathogenesis of several non-infectious inflammatory disorders [19,20,21,22,23,24].

Most importantly, NETs may exert significant pro-fibrotic properties by presenting distinctly bioactive inflammatory cytokines, such as IL-17A, which can mediate fibrotic remodeling. In this context, experimental data from patients with an intermediate/high fibrotic risk have shown that NETs expressing functional IL-17A can enhance the proliferation and activation status of tissue fibroblasts [19,25,26]. Fibroblasts have multiple biological roles, including the production of matricellular proteins, such as cellular communication network factor 2 (CCN2), and the release of collagen [25], subsequently resulting in fibrotic damage. As such, NETs have been implicated in the pathogenesis of asthma in adults. Indeed, both NETs and eosinophil extracellular traps (EETs) seem to be implicated in persistent asthma [27]; however, the aspects of these mechanisms remain understudied. In particular, the protein profile of NET structures, which is determined by the disease microenvironment, is not known, and NETosis in severe childhood asthma remains a scientific puzzle.

Herein, we identify neutrophils/NETs expressing IL-17A as a main component of asthma exacerbation (hereafter mentioned as asthma) in children. This study unveils the inflammatory effect of IL-17A-enriched asthma NETs on human lung fibroblasts, possibly correlating them with the fibrotic events of the disease. 

## 2. Materials and Methods

### 2.1. Patients and Sample Collection

Serum samples were obtained from 29 asthmatic children (asthma serum) and 29 healthy children (control serum). Asthma sera were collected from asthmatic children during disease exacerbation, possibly triggered by allergic sensitization or exposure to aeroallergens. Asthmatic children with a possible bacterial infection or viral pneumonia were excluded [28]. Control sera were collected from children that visited the outpatient unit of the Pediatric Department of the University Hospital of Alexandroupolis, Greece, for a sports medical certificate. For serum isolation, peripheral whole blood was collected in proper blood collection tubes (BD Vacutainer^®^ Clot Activator Tubes, Becton, Dickinson and Company, Franklin Lakes, NJ, USA) and centrifuged at 500× *g* for 15 min. All samples were temporarily stored at −20 °C until further analysis [19,26].

Clinical data collection, patient characterization and venipuncture were performed by authorized clinicians in accordance with current guidelines (Table 1). Informed consent was obtained from all subjects involved in the study and was signed by both parents. All procedures complied with the Declaration of Helsinki and had the approval of the Ethics Committee of the University Hospital of Alexandroupolis, Greece (Ethics Committee protocol number 17,788).

To evaluate the allergy status of children, serum IgE concentration was determined using electrochemiluminescence (ECL) (Roche Elecsys, Basel, Switzerland). The serum IgE levels are age-dependent, and the limit of normalcy for IgE levels is 150 IU/mL in school-aged children [29,30]. In this study, the IgE values in subjects with asthma were significantly elevated compared to the controls (*p* < 0.0001) (Table 1).

### 2.2. Neutrophil Isolation

Healthy individual (HI) neutrophils were isolated from heparinized peripheral blood and collected in appropriate blood collection tubes (BD Vacutainer^®^ Heparin Tubes, Becton, Dickinson and Company, Franklin Lakes, NJ, USA), by performing Histopaque double-gradient density centrifugation (11,191 and 10,771, Sigma-Aldrich, St. Louis, MO, USA). In line with the manufacturer’s instructions, samples were centrifuged at 700× *g* for 30 min at 20–25 °C. Thereafter, neutrophils were centrifuged at 200× *g* for 12 min at 20–25 °C in the presence of 1× Dulbecco’s phosphate-buffered saline solution (1× PBS) [31]. The purity of the isolated neutrophil population exceeded 98%.

### 2.3. Human Embryonic Lung Fibroblast (HELF) Culture

Human embryonic lung fibroblasts (HFL-1, Cat#: CCL-153, American Type Culture Collection, Manassas, WV, USA) were cultured at 37 °C with 5% CO_2_ in culture medium, consisting of low-glucose Dulbecco’s Modified Eagle’s Medium (DMEM, Thermo Fisher Scientific, Waltham, MA, USA), 10% *v*/*v* fetal bovine serum (FBS, Thermo Fisher Scientific, Waltham, MA, USA), 100 U/mL Antibiotic-Antimycotic (Biosera, Cholet, France) and 5% *v*/*v* MEM non-essential amino acids solution (Thermo Fisher Scientific, Waltham, MA, USA). Cell culture passaging was performed using 0.05% Trypsin-EDTA (Thermo Fisher Scientific, Waltham, MA, USA) [25,32,33]. All experiments were carried out with HELFs culture at passages 25–29.

### 2.4. Stimulation and Inhibition Studies

#### 2.4.1. Neutrophils

Isolated HI neutrophils were incubated at 37 °C with 5% CO_2_ in Roswell Park Memorial Institute 1640 medium (RPMI-1640, Thermo Fisher Scientific, Waltham, MA, USA), supplemented with 2% *v*/*v* FBS. Subsequently, they were stimulated in vitro for 3 h at 37 °C with 5% CO_2_, using 4% asthma serum or control serum (untreated condition) [17,21]. Immunofluorescence and NET structure generation were performed for experimental readouts. Same studies were carried out (70 min, 37 °C, 5% CO_2_) for qPCR analysis. HI neutrophils stimulated with control serum were used as control group in qPCR experiments.

#### 2.4.2. HELFs

Upon reaching appropriate confluency, HELFs were stimulated at 37 °C with 5% CO_2_, using 20% in vitro-generated IL-17A-enriched asthma exacerbation NETs (asthma NETs) (DNA concentration: 0.5 μg/mL) in order to examine the interaction between HELFs and asthma NETs. To evaluate the NET scaffold and NET-derived IL-17A effect on fibroblast activation, two types of inhibitions were performed. NET scaffold dismantling was accomplished via NET pre-incubation using 1 U/mL recombinant DNase I (Takara Bio, Shiga, Japan), whereas IL-17A was neutralized using 10 mg/mL IL-17A antibody (R&D, Minneapolis, MN, USA). Both inhibitions were performed at 37 °C for 30 min [26]. The effect of NETs on HELFs’ fibroblast dynamic was investigated via both collagen measurement and wound healing assay. Unstimulated HELFs were used as negative control.

### 2.5. NET Structure Generation and Collection

For NET generation, 2 × 10^6^ HI neutrophils, cultured in RPMI in a 6-well plate (SPL Life Sciences, Kyonggi-do, Republic of Korea), were stimulated with 6% asthma serum or control serum for 3 h (37 °C, 5% CO_2_). Upon completion of stimulation, the culture supernatant was carefully removed, and cells were washed with pre-warmed RPMI. An amount of 1 mL fresh RPMI was added to each well, followed by vigorous plate shaking to collect NETs. The supernatant was collected and centrifuged at 20× *g* for 5 min. NETs were isolated in the supernatant phase and stored at −20 °C until further use [34,35]. Quantification of NETs was assessed via SYTOX Green Staining (Invitrogen, Waltham, MA, USA) and citrullinated histone 3 (H3Cit) ELISA (Cayman Chemical, Ann Arbor, MI, USA) according to the manufacturer’s instructions. The supernatant from HI neutrophils stimulated with control serum served as negative control.

### 2.6. Collagen Measurement

Following the abovementioned HELF stimulation/inhibition studies, culture supernatants from 6-well plates were collected after 24 h for collagen measurement, using Sircol Soluble Collagen Assay kit (Biocolor Life Science Assays, Co Antrim, UK). Supernatants were centrifuged (13,000× *g*, 15 min), incubated (overnight, 4 °C) using collagen isolation and concentration reagent (kit included) and further processed according to the manufacturer’s protocol, to measure soluble collagen (type I-V).

### 2.7. Migration/Wound Healing Assay

To evaluate the migratory/healing capacity of HELFs, cells were seeded in a 24-well plate (Ibidi, Gräfelfing, Germany). When HELFs were 90% confluent, stimulation/inhibition studies were performed and healing was evaluated after 20 h of incubation [33,36]. Assessment of wound healing was achieved with both immunofluorescence and May–Grünwald–Giemsa (MGG) stain. Assay was performed by following the manufacturer’s instructions and recommendations. 

### 2.8. H3Cit ELISA

Citrullinated histone 3 was measured in asthma and healthy serum samples using H3Cit ELISA (Cayman Chemical, Ann Arbor, MI, USA) in accordance with the manufacturer’s instructions.

### 2.9. IL-17A ELISA

IL-17A ELISA (R&D Systems, Minneapolis, MN, USA) was utilized to measure IL-17A concentration in asthma and healthy serum samples and in in vitro-generated NETs. The assay was performed following the manufacturer’s protocol. 

### 2.10. RNA Extraction, cDNA Synthesis and qPCR

HI neutrophils stimulated with asthma serum were processed for RNA extraction, cDNA synthesis and qPCR, as formerly detailed [19,25,37]. To verify *IL-17A* expression in asthma-serum-treated neutrophils in vitro, expression of *IL-17A* (forward primer: 5′TGGTGTCACTGCTACTG3′, reverse primer: 5′CATTGCGGTGGAGATTC3′) was evaluated via qPCR. *GAPDH* (forward primer: 5′GGGAAGCTTGTCATCAATGG3′, reverse primer: 5′CATCGCCCCACTTGATTTTG3′) was utilized to normalize *IL-17A* expression, following the housekeeping gene method of normalization. qPCR was performed, and the 2^−ΔΔCt^ method was applied for data analysis [38].

### 2.11. Immunofluorescence

#### 2.11.1. Neutrophils

Isolated HI neutrophils were seeded in a 24-well plate (SPL Life Sciences, Kyonggi-do, Republic of Korea) on poly-l-lysine coverslips (Neuvitro, Camas, WA, USA). After 3 h of stimulation/inhibition, cells were fixed with 10% formaldehyde (Biognost, Zagreb, Croatia) (30 min, 4 °C), blocked with 6% normal goat serum (OriGene, Rockville, MD, USA) in 1× PBS and incubated with primary antibody solution consisting of 1:100 anti-neutrophil elastase (Abcam, Cambridge, UK) and 1:50 anti-IL-17A (R&D System, Minneapolis, MN, USA) antibodies in blocking solution for 1 h at room temperature (RT). Then, 1× PBS washes and incubation (1 h, RT) in secondary antibody solution were performed. CF488A and CF594 (Biotium, Fremont, CA, USA), diluted as recommended by the manufacturer, were used as secondary antibodies. As a final step, cells were incubated in DAPI solution (Sigma-Aldrich, St. Louis, MO, USA) for 2 min and 30 s at RT and mounted on microscope slides (Knittel Glass, Braunschweig, Germany) using ProLong Diamond Antifade Mountant (Thermo Fisher Scientific, Waltham, MA, USA) [23]. Sample visualization was performed on a Nikon ECLIPSE Ti2 Inverted Microscope (Nikon, Melville, NY, USA) using a 40× oil lens (1.30 NA), and image acquisition was achieved using NIS-Elements software (Nikon, Melville, NY, USA). All appropriate isotype controls were prepared. Confocal images were analyzed using Fiji software version 2.9.0 [39]. 

The percentage of NET release was determined through the measurement of 200 cells in a blinded experimental procedure [19].

#### 2.11.2. HELFs

Following the migration/wound healing assay, seeded HELFs were fixed, blocked and stained without being removed from the 24-well plate, using the abovementioned immunofluorescence protocol. As primary and secondary antibodies, anti-CCN2 (Santa Cruz Biotechnology, Dallas, TX, USA) (1:100 dilution) and CF488A (Biotium, Fremont, CA, USA) were used, respectively. Stained cells were simultaneously stained for DAPI and mounted with a non-hardening mounting medium (Ibidi, Gräfelfing, Germany) [33]. Images were acquired using a 10× air lens (0.45 NA) on a Nikon ECLIPSE Ti2 Inverted Microscope (Nikon, Melville, NY, USA) using NIS-Elements software (Nikon, Melville, NY, USA). Necessary isotype controls were used for this experiment. Confocal images were processed using Fiji software version 2.9.0 [39].

### 2.12. MGG Stain

Visualization of HELFs’ migrating capacity in the migration/wound healing assay was achieved through MGG staining. Cells were incubated with May–Grünwald stain for 5 min at RT. Subsequently, excessive stain was washed away with water, following a 20 min incubation with 1:10 diluted Giemsa stain. Cells were washed with water and observed under an upright light microscope with a 4× air lens. The final images were produced using Fiji software version 2.9.0 [39].

### 2.13. Statistical Analysis

The comparisons between two independent groups, i.e., patients with asthma and HIs (controls) or in vitro neutrophil studies, were performed with the use of the Mann–Whitney *U* test. Kruskal–Wallis test, followed by Dunn’s test, was used for comparisons among more groups. Spearman’s rank correlation coefficients were used to describe bivariate correlations. The level of statistical significance was set to *p* < 0.05. Means are accompanied by their 95% confidence intervals (CIs). Statistical analysis was performed using GraphPad Prism version 9 (GraphPad Software, Inc., San Diego, CA, USA).

## 3. Results

### 3.1. Increased Levels of Circulating NETs in Patients with Asthma Exacerbation

**Figure 1:**
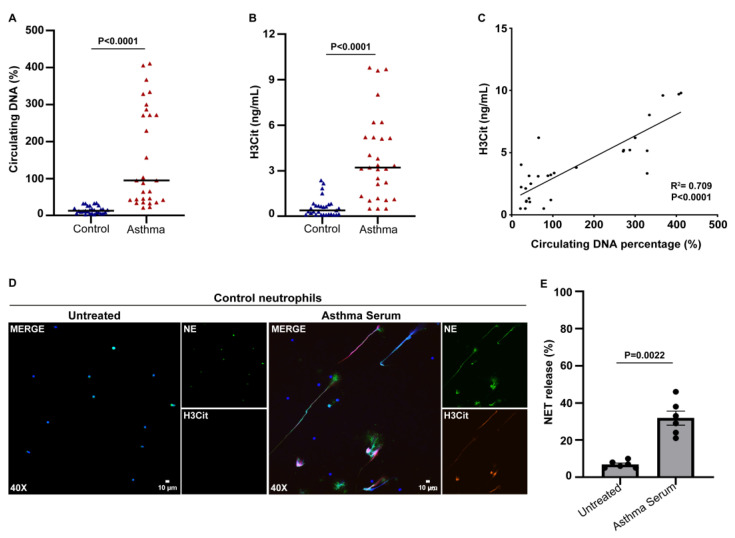
Markers of neutrophil extracellular traps (NETs) in the circulation of patients with asthma exacerbation and control neutrophils stimulated with asthma exacerbation serum. Levels of (**A**) circulating DNA and (**B**) H3Cit representing NET release in asthma serum compared to healthy individuals (controls) (*n* = 29 subjects per group). (**C**) Correlation between circulating DNA and H3Cit levels. Spearman’s r2 values are shown. (**D**) Fluorescence microscopy images showing NE/H3Cit staining (blue, DAPI; green, NE; red, H3Cit; original magnification, 400×) and (**E**) percentage of NET release (*n* = 6 independent experiments), as assessed via immunofluorescence, in control neutrophils incubated with asthma serum. Untreated condition refers to control neutrophils stimulated with sera from healthy subjects (control serum). For (**A**,**B**,**E**), data are shown as mean ± SD, Mann–Whitney *U* test (2 tailed). For (**D**), a representative example of 6 independent experiments is shown. All conditions were compared to controls/untreated (statistically significant: *p* < 0.05).

Studies have implied that there is an association between neutrophil-dominated inflammation and the severity of asthma [40,41]. Excessive NETs can influence the pathogenesis of numerous inflammatory disorders, including adult asthma. For this reason, we measured the relative levels of cell-free circulating DNA in the sera of asthma patients compared to the control group (Figure 1A). However, since cell-free DNA can originate from cell populations other than neutrophils, we engaged an ELISA, enabling the measurement of H3Cit, which is a well-defined circulating marker of NETs [37]. The H3Cit levels were significantly increased in patients with asthma compared to the controls (Figure 1B). Furthermore, there was a strong correlation between the cell-free DNA and H3Cit values (Figure 1C).

In an effort to support our ex vivo observations, and since distinct inflammatory mediators can enhance the release of NETs [42,43], the neutrophils isolated from healthy individuals (control neutrophils) were stimulated with asthma serum samples, representing the inflammatory disease microenvironment. We found that the asthma-serum-treated control neutrophils formed NETs, as assessed via immunofluorescence microscopy (Figure 1D) and H3Cit ELISA on isolated NETs (Figure 1E). Collectively, our findings show that the patients exhibited high serum levels of NETs during asthma exacerbation, while the inflammatory disease microenvironment mediated in vitro NET generation.

### 3.2. Serum from Asthma Patients during Exacerbation Induces the Release of NETs Carrying IL-17A

**Figure 2:**
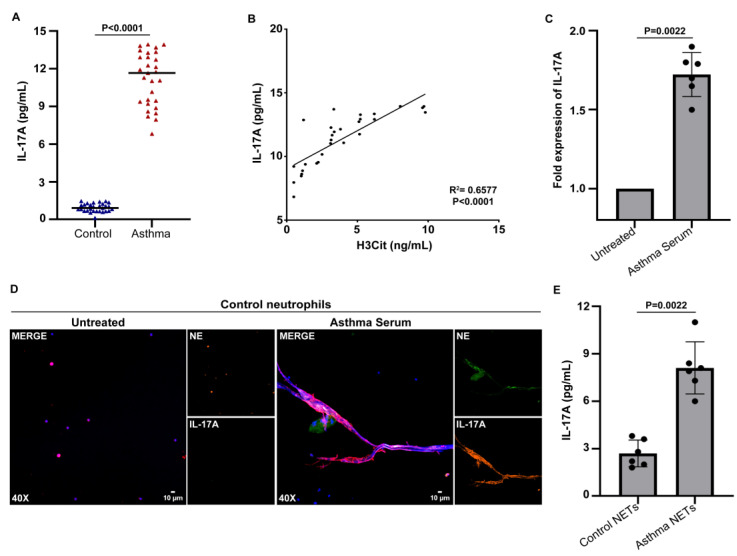
Control neutrophils stimulated with asthma exacerbation serum release neutrophil extracellular traps (NETs) expressing interleukin (IL)-17A. (**A**) Levels of IL-17A in asthma serum compared to healthy individuals (controls) (*n* = 29 subjects per group). (**B**) Correlation between H3Cit, representing NET release, and IL-17A levels in asthma serum. Spearman’s r2 values are shown. (**C**) *IL-17A* mRNA expression in control neutrophils treated with asthma serum, as assessed via qPCR (*n* = 6 independent experiments). (**D**) Fluorescence microscopy images showing IL-17A/NE staining (blue, DAPI; green, IL-17A; red, NE; original magnification, 400×) in control neutrophils incubated with asthma serum. For (**C**,**D**), untreated condition refers to control neutrophils stimulated with serum from healthy subjects (control serum). (**E**) IL-17A levels in in vitro-generated NET structures (*n* = 6 independent experiments). NETs were obtained via control neutrophils incubated with asthma serum (asthma NETs) or serum from healthy subjects (control NETs). For (**A**,**C**,**E**), data are shown as mean ± SD, Mann–Whitney *U* test (2 tailed). For (**D**), a representative example of 6 independent experiments is shown. All conditions were compared to controls/untreated (statistically significant: *p* < 0.05).

Chronic low-grade inflammation and fibrotic manifestations are key pathological features of asthma [44,45]. Moreover, IL-17A is a pro-inflammatory cytokine implicated in asthma pathogenesis [46], whereas IL-17A-enriched NETs can exert a potent fibrotic role [19,25,26]. Therefore, the levels of IL-17A were evaluated in the sera of asthma patients using an ELISA assay and were found to be markedly increased in the patient group compared to the control group (Figure 2A). In addition, there was a positive correlation between the H3Cit and IL-17A levels in the asthma serum, proposing a link between NET formation and fibrosis in asthma (Figure 2B).

To further verify the immunofibrotic aspect of the disease, in vitro stimulations were deployed. As indicated by real-time quantitative PCR (qPCR), the serum from the asthmatic patients was able to induce *IL-17A* expression in the control neutrophils (Figure 2C). Given that the pathogenesis of neutrophil-driven disorders is essentially determined by the protein composition of NETs [47], we then tested whether IL-17A is externalized on NETs, formed by control neutrophils, upon treatment with asthma serum. Indeed, we found that asthma-serum-treated control neutrophils efficiently generated IL-17A-enriched NETs, as indicated via the immunofluorescence microscopy (Figure 2D) and IL-17A ELISA on isolated NETs (Figure 2E).

### 3.3. IL-17A-Enriched NETs Activate Human Lung Fibroblasts toward Collagen Production

The association between fibroblast hyperactivation and asthma is well established [45]; however, the underlying mechanisms leading to the fibrotic aspects of the disease are not well defined. In the same context, the crosstalk between NETs and fibroblasts seems to deregulate fibroblast function based on previous data [19,25].

**Figure 3:**
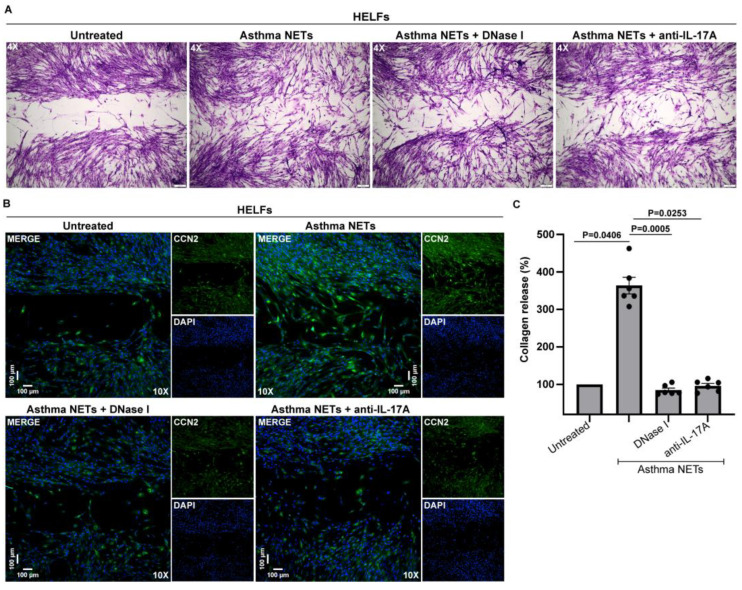
Human embryonic lung fibroblasts (HELFs) acquire a pro-fibrotic phenotype upon treatment with in vitro-generated asthma exacerbation NETs. HELFs were incubated with NETs released from control neutrophils upon stimulation with asthma serum (asthma NETs). (**A**) Migration/wound healing potential (original magnification, 40×) in HELFs stimulated with asthma NETs, assessed via light microscopy. (**B**) Fluorescence microscopy images showing CCN2 staining (blue, DAPI; green, CCN2; original magnification, 100×) and migration/wound healing capacity in HELFs treated with asthma NETs and (**C**) production of collagen (*n* = 6 independent experiments). To hinder IL-17A signaling, asthma NETs were pre-incubated with a neutralizing antibody against IL-17A. DNase I was used to dismantle NETs. For (**A**,**B**), representative examples of 4 independent experiments are shown. For (**C**), data are shown as mean ± SD, Kruskal–Wallis test. All conditions were compared to untreated (statistically significant: *p* < 0.05).

Therefore, we deployed a co-culture system between HELFs and disease NETs. HELFs were stimulated with NETs, generated by the in vitro exposure of control neutrophils to asthma serum (hereafter named asthma NETs). Concurrently, we detected that mesenchymal cells enhanced their migratory/healing potential (Figure 3A,B), CCN2 protein expression (Figure 3B) and collagen production (Figure 3C). On the other hand, asthma NETs, pre-incubated with DNase I, led to a lessened migratory/healing capacity, CCN2 expression and collagen release in the HELFs (Figure 3A–C). Similar findings were also obtained upon the treatment of NETs with a neutralizing antibody against IL-17A (Figure 3A–C), underlining the pro-inflammatory dynamic of asthma NETs in the fibrotic aspect of HELFs. Taken together, our findings indicate that IL-17A-enriched NETs, induced by the asthma microenvironment, may promote the HELFs’ dysfunction, switching them to a pro-fibrotic state.

## 4. Discussion

This study links neutrophils/NETs with lung immunofibrosis in childhood asthma exacerbation. The microenvironment of asthma triggers the release of NET-bound IL-17A with a pro-fibrotic activity. Particularly, the inflammatory milieu of asthma exacerbation has arisen as an inducer of the NET mechanism, whereas IL-17A, which is expressed on NET structures, promotes collagen production by lung fibroblasts.

Asthma is a complex disease with a heterogenous profile [48,49,50]. However, over the past two decades, our understanding of asthma pathogenesis has significantly evolved. Remarkably, recent data focusing on severe non-eosinophilic forms of asthma have shown that neutrophils, inflammatory cytokines and/or mesenchymal cells may play a decisive role in the severity and progression of the disease [7,51,52,53,54,55]. In the same context, studies have supported that disease progression in adulthood may be predisposed by repeated exacerbations of asthma in childhood [56,57].

Neutrophils have been traditionally recognized as essential players in acute, bacterial and fungal infections. Nevertheless, recent data have indicated an increased neutrophilic response in severe asthma, especially in children [58,59]. Based on several studies, elevated neutrophil counts are an important indicator of asthma severity, since their accumulation is linked to a heightened risk of exacerbations, a rapid decline in lung function and a lower response to conventional therapies [4,60,61]. 

Moreover, neutrophil products, for example, NETs, have been associated with inflammatory and fibrotic responses in certain disorders, including adult asthma [19,25,33,40]. Previous findings have shown that NETs can drive airway obstruction and inflammation in adult asthmatic patients [52,62]. However, there are no data linking the mechanism of NETs to the pathogenesis of childhood asthma. Our findings demonstrate that control neutrophils exposed to asthmatic serum, derived from pediatric patients during asthma exacerbation, release pro-inflammatory NETs (asthma NETs), bridging the NET pathway to disease exacerbations.

In the same context, studies have demonstrated that the mechanism of NETs enhances inflammatory responses by promoting the release of pro-inflammatory proteins, which are capable of recruiting and activating distinct cell populations [19,23,26,63]. Importantly, experimental data have underlined that neutrophils can form NETs, which are qualitatively different, within different disease microenvironments [47,64,65]. On the other hand, the protein load of asthma NETs in adults remains understudied. Our findings assess that asthma NETs, released upon the stimulation of control neutrophils with the inflammatory disease microenvironment, are enriched with IL-17A, indicating a possible link between this pro-inflammatory cytokine and childhood asthma pathogenesis. Indeed, our data are consistent with those of previous studies, showing that IL-17A is a key mediator that is implicated in neutrophilic responses in adult asthma. In addition, there are studies reporting that elevated levels of IL-17A are associated with severe asthma and increase asthma exacerbations, and thus, IL-17A might shape the progression of the disease [46,66].

Furthermore, the role of fibroblasts and fibrosis in asthma is gaining widespread acceptance. Airway remodeling, including subepithelial fibrosis, is a characteristic feature of chronic asthma. Studies have demonstrated that asthmatic children exhibit abnormal reticular basement membrane (RBM) thickening early in the disease. This clinical feature is explained by interstitial collagen deposition, which is reflected in the increased fibril-to-matrix ratio [67]. The data derived from adult patients with moderate to severe disease have recently indicated that this aspect of airway remodeling may be regulated by gene expression alterations and epigenetic modifications [68].

The involvement of fibroblasts, which are the main source of extracellular matrix, in this remodeling process, as well as their close interaction with various immune cells, was revealed by recent studies [7,25,69]. Various inflammatory microenvironments, including the asthmatic one, enhance the differentiation of fibroblasts into myofibroblasts, leading to collagen release and contributing to airway fibrosis [25,70]. Interestingly, evidence has shown that this process can be mediated by NETs [19,25,71] and/or several pro-inflammatory cytokines [10,72]. However, the exact mechanisms underlying these interactions in childhood asthma remain poorly understood. Our findings indicate that HELFs acquire a potent pro-fibrotic phenotype upon treatment with IL-17A-enriched NETs. Moreover, NET dismantling with DNase I or IL-17A blocking on NET structures markedly reduces the fibrotic potential of HELFs, suggesting that neutrophils/NETs can be considered both markers of disease severity and active contributors to asthma pathogenesis.

There is currently a pressing need for effective biomarkers in childhood asthma. Although several biomarkers were tested in the context of childhood asthma, most of them are less useful in neutrophilic asthma due to a lack of eosinophilic inflammation [73,74,75]. The enhancement of childhood asthma management demands the development of novel biomarkers for the purpose of differential diagnosis and for the characterization of distinct asthma endotypes. Hence, our observations, which are consistent with those of other recent studies [76,77], suggest that neutrophilic proteins released by activated neutrophils/NETs could be considered as potential biomarkers of neutrophilic inflammation in childhood asthma. 

Therapies for neutrophilic asthma in children remain a challenge. The mainstay of therapy continues to be inhaled corticosteroids, which are often used in combination with long-acting beta agonists. However, the response to these treatments is unpredictable and often suboptimal, particularly in severe steroid-resistant cases [78,79]. Our data show that NETs and specific cytokines can be considered as promising treatment targets. Experiments in an animal asthma model have shown that blocking IL-17A or its receptor can reduce neutrophilic inflammation [80]. However, clinical trials in humans are still in their early stages and have yet to show clear benefits in asthma. In addition, targeting NETs can also represent a novel therapeutic strategy for neutrophilic asthma. Indeed, drugs that are capable of dismantling NETs or inhibiting their formation are being investigated, but further research is needed to determine their efficacy and safety [81,82,83,84].

In conclusion, our study provides a deeper understanding of the complex interactions between neutrophils/NETs and fibroblasts in the context of neutrophil-mediated fibrosis in childhood asthma exacerbation. NETs, neutrophilic proteins (e.g., neutrophil elastase) and NET-specific immunoproteins (e.g., IL-17A) hold promise as biomarkers of asthma and for the development of more effective therapies in the future.

## Figures and Tables

**Table 1:** Baseline characteristics of study population.

	Asthma n = 29	Controls n = 29	*p*-Value
**Sex** male, (%)	18 (62%)	16 (55.2%)	0.182
**Age** (years), mean (±SD)	8.9 ± 4.7	10.9 ± 5.7	0.071
**Comorbidities**		-	
**AD** n (%)	12 (41.4%)	-	
**FA** n (%)	6 (20.7%)	-	
**AR** n (%)	14 (48.3%)	-	
**Laboratory data**			
**Ig E** IU/mL mean (±SD)	582.7 ± 188.4	103.13 ± 16.7	0.0001
**WBC** count (1000/mm^3^)	8.9	7.4	0.21
**Neutrophil** count	62%	58.4%	0.59
**Eosinophil** count	8.4%	3.2%	0.0001
**CRP** mg/dL mean (±SD)	0.95 (±0.84)	0.65 (±0.41)	0.44
**Asthma medication**			
**Inhaled SABA frequency** n (%)			
**None**	2 (6.9%)	-	
**<1/month**	20 (68.9%)	-	
**≥1/month**	7 (24.1%)	-	
**Asthma severity** n (%)			
**Intermittent**	8 (27.5%)	-	
**Mild persistent**	9 (31%)	-	
**Moderate or severe persistent**	12 (41.4%)	-	
**Inhaled corticosteroid grade**			
**None**	4 (13.8)	-	
**Low dose**	13 (44.8%)	-	
**Medium or high dose**	12 (41.4%)	-	

AD, atopic dermatitis; FA, food allergy; AR, allergic rhinitis; WBC, white blood cell; SABA, short-acting beta agonist; CRP, C reactive protein.

## Data Availability

The data presented in this study are available within the article. More detailed data, presented in this study, are available on request from the corresponding author.
